# Xp11.2 Duplication in Females: Unique Features of a Rare Copy Number Variation

**DOI:** 10.3389/fgene.2021.635458

**Published:** 2021-04-14

**Authors:** Márta Czakó, Ágnes Till, Judith Zima, Anna Zsigmond, András Szabó, Anita Maász, Béla Melegh, Kinga Hadzsiev

**Affiliations:** ^1^Department of Medical Genetics, Medical School, University of Pécs, Pécs, Hungary; ^2^Szentágothai Research Centre, Pécs, Hungary

**Keywords:** Xp11.23p11.22 duplication, array CGH, X-inactivation, speech and language delay, regression

## Abstract

Among the diseases with X-linked inheritance and intellectual disability, duplication of the Xp11.23p11.22 region is indeed a rare phenomenon, with less than 90 cases known in the literature. Most of them have been recognized with the routine application of array techniques, as these copy number variations (CNVs) are highly variable in size, occurring in recurrent and non-recurrent forms. Its pathogenic role is not debated anymore, but the information available about the pathomechanism, especially in affected females, is still very limited. It has been observed that the phenotype in females varies from normal to severe, which does not correlate with the size of the duplication or the genes involved, and which makes it very difficult to give an individual prognosis. Among the patients studied by the authors because of intellectual disability, epilepsy, and minor anomalies, overlapping duplications affecting the Xp11.23p11.22 region were detected in three females. Based on our detailed phenotype analysis, we concluded that Xp11.23p11.22 duplication is a neurodevelopmental disorder.

## Introduction

The technical possibilities of copy number variation (CNV) detection were significantly improved in the last 15 years, as a result the abnormality causing the pathological phenotype and the mechanism of their development have become known in many diseases (Zhang et al., 1997, 2009; Lupski, 1998; Marshall et al., 2008; Carvalho and Lupski, 2016). Chromosome microarray has become a first-line investigation tool, particularly in patients with intellectual disability (ID), multiple malformations, epilepsy and autism (Miller et al., 2010). In syndromic forms of ID, where morphological abnormalities, behavioral disorders, seizures, and abnormal growth are associated with the disease, the group showing X-linked inheritance is remarkable. This is not surprising considering that based on new data published in the last decade; we know that almost twice as many genes are associated with X-linked mental retardation as thought before (Neri et al., 2018). Among ID patients, the proportion of males is slightly higher (1, 3:1 male to female), 5–10% of the cases shows X-linked inheritance (Froyen et al., 2008). Their studies revealed the role of more than 100 genes located on chromosome X, and a number of CNVs have been described (Froyen et al., 2007; Neri et al., 2018). While among pathogenic CNVs detected on autosomes deletions occur at a higher rate, there are much more duplications occurring on chromosome X (Ropers, 2006; Whibley et al., 2010; Lubs et al., 2012; Vulto-van Silfhout et al., 2013).

The duplication affecting the Xp11.23p11.22 region is unique even among these specific CNVs of X chromosome. It is very rare occurring in both genders (Kokalj Vokac et al., 2002; Bonnet et al., 2006; Froyen et al., 2008, 2012; Monnot et al., 2008; Giorda et al., 2009; Zou and Milunsky, 2009; Holden et al., 2010; Honda et al., 2010; Broli et al., 2011; Chung et al., 2011; Edens et al., 2011; El-Hattab et al., 2011; Flynn et al., 2011; Nizon et al., 2014; Evers et al., 2015; Grams et al., 2015; Moey et al., 2016; Orivoli et al., 2016; Arican et al., 2018; Wang et al., 2020). Inherited and *de novo* forms are known, all *de novo* Xp11.23 duplications in which parent of origin has been determined have been paternally inherited (Deng et al., 1990). Generally, duplications of the X chromosome in females are often asymptomatic because X-inactivation process silences the chromosome carrying the duplication (Van den Veyver, 2001). However, in case of Xp11.23p11.22 duplications, the opposite is true: in females with skewed X-inactivation the X chromosome carrying the wild-type allele is silenced, while the abnormal one is active in the majority of the cells. Therefore, females with random X-inactivation develop a milder phenotype.

Based on the cases reported so far, the phenotypic features which develop in males and females affected by duplication of Xp11.23p11.22 are very similar (moderate to severe ID, significant delay of speech development, very specific pattern observed on electroencephalography (Broli et al., 2011) with or without seizures manifestation, and dysmorphic facial features), which is very thought provoking in terms of the pathomechanism. While in boys, the pure increase in gene dose caused by the extra copy may explain the symptoms, the gene dosage assessment in girls is much more complicated due to skewed or random X-inactivation (Bonnet et al., 2006; El-Hattab et al., 2011; Nizon et al., 2014; Orivoli et al., 2016; Wang et al., 2020).

The relationship between the genes affected by the Xp11.23p11.22 duplication and the phenotype is also very complex. Non-recurrent duplications of 0, 3–55 Mb in size have been reported in addition to the recurrent form of about 4.5 Mb, therefore the genes involved in the individual patient’s duplications may be quite different. Patients with this copy number alteration, which have become known so far, show surprisingly similar symptoms despite the variability in CNV size: the resulting symptoms do not correlate with size and gene content of the affected genomic regions (Giorda et al., 2009; Zou and Milunsky, 2009; Flynn et al., 2011; Arican et al., 2018).

**FIGURE 1:**
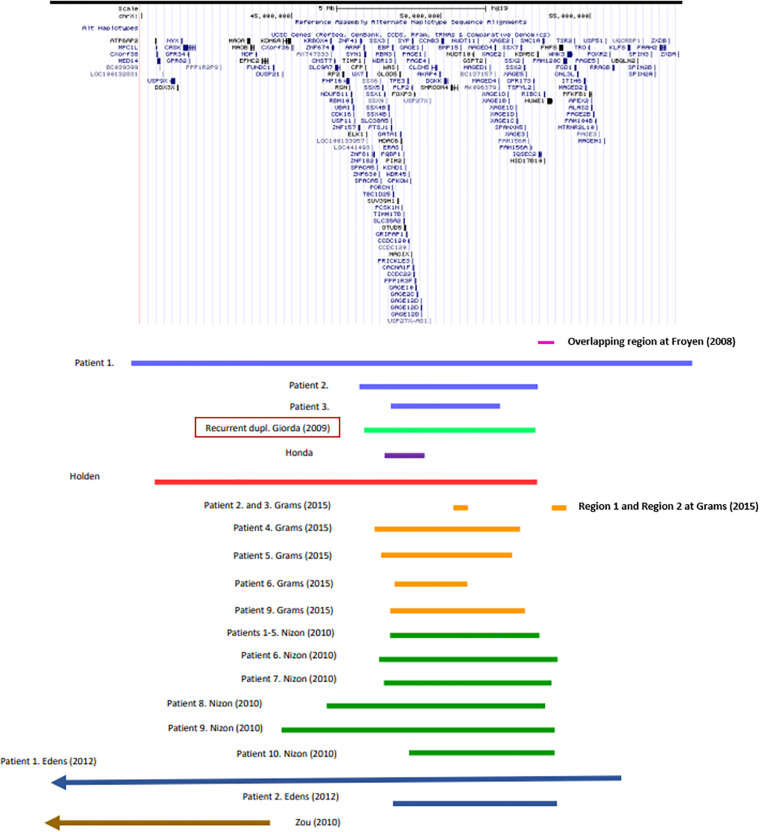
Recurrent and non-recurrent Xp11.22p11.23 duplications of the patients listed in Supplementary Table 1 (UCSC Genome Browser on Human Feb. 2009 (GRCh37/hg19) Assembly).

In particular, genotype-phenotype analysis of cases with non-recurrent duplication offered opportunity to analyze the relationship between shared symptoms and affected genomic regions. Several studies have found association between certain genes and symptoms (Table 1), however, the individual studies did not examine exactly the same phenotypic traits, which makes the correlation difficult. In a study of six families, Froyen et al. (2008) isolated a minimal overlapping region. Functional analysis of the genes involved identified a causal relationship between elevated gene dosage and intellectual disability only in case of the *HUWE1* gene (Froyen et al., 2012). Grams et al. (2015) based on their own analyses and the previously published cases with duplications of Xp11.2 highlighted the importance of two smaller subregions. One of them (Region 1) contains the *SHROOM4* and *DGKK* genes, the other (Region 2) is the same as that was examined by Froyen et al., in which in addition to *HUWE1, KDM5C*, and *IQSEC2* are included as candidate genes (Table 1 and Figure 1). Taken together, based only on the genes with extra copy, the expected phenotype cannot be predicted in individual patients.

**TABLE 1:** Genes involved in Xp11.22p11.23 duplications.

**Gene (OMIM #)**	**Function**	**Associated symptoms**
*TM4SF2* (300096)	Tetraspanin 7, control of neurite outgrowth	Intellectual disability (ID)
*ZNF41* (314995)	Zinc finger protein 41	ID, speech delay
*ZNF81*	Zinc finger protein 81	ID
*SYN1* (313440)	Synapsin I, regulation of neuronal development	seizures, learning difficulties, behavioral abnormalities
*FTSJ1* (300499)	Homolog of Escherichia coli RNA methyltransferase FtsJ/RrmJ; takes part in the regulation of translation	ID, agressive behavior (obesity, macrocephaly)
*PQBP1* (300463)	Transcriptional activator; overexpression of *PQBP1* suppressed the cell growth (stress susceptibility)	ID, microcephaly, neuronal dysfunction, short stature, spasticity
*HDAC6* (300272)	Histone deacetylase 6	ID
*ATP6AP2* (300423)	Renin/prorenin receptor precursor	Seizures, ID, motor and speech delay
*CASK* (300172)	Calcium/calmodulin-dependent serine protein kinase	ID, microcephaly, brain malform
*ZNF674* (300573)	Kruppel-type zinc finger protein; transcriptional regulator	ID, learning difficulties, disturbances of adaptive behavior
*MAOA* (309850)	Monoamine oxidase localized in the outer mitochondrial membrane	ID and agressive behavior in males
*BCOR* (300485)	BCL-6 corepressor; key transcriptional regulator during early embryogenesis in eyes and central nervous system	Microphthalmia (syndromic, type 2), low weight, short stature, teeth anomalies, heart failure, seizures, scoliosis, ID, motor delay
*NDP* (300658)	A norrin precursor; neuroectodermal cell-cell interaction	Vitreoretinopathy, psychosis, growth failure, seizures
*NYX* (300278)	A nyctalopin precursor	Myopia, hyperopia, nystagmus, reduced visual acuity
*RP2* (300757)	Stimulates the GTPase activity of tubulin	XL-retinitis pigmentosa 2
*SYP* (313475)	Synaptophysin; an integral membrane protein that regulates synaptic vesicle endocytosis	ID, epilepsy
*BMP15* (300247)	Bone morphogenetic protein (BMP) 15; oocyte-specific growth and differentiation factor	Abnormal growth parameters; early puberty
*KDM5C* (314690)	Lysine-specific demethylase 5C; transcriptional repressor	ID, autism spectrum disorder, spastic paraplegia
*HUWE1* (300697)	HECT, UBA, and WWE domains-containing protein 1; E3 ubiquitin ligase,	ID, neuronal development, proliferation, synaptogenesis
*PHF8* (300560)	Zinc finger protein 422	ID
*FGD1* (300546)	FYVE, RhoGEF, and PH domains-containing protein 1	ID
*SLC35A2* (314375)	Solute carrier family 35 (UDP-galactose transporter), member 2	Abnormal galactosylation in neurons; seizures
*SHROOM4* (300579)	SHROOM family member 4, Stocco dos Santos XLMR-syndrome; influences cytoskeletal architecture	Severe ID, delayed/no speech, seizures, hyperactivity
*KCND1* (300281)	potassium voltage-gated channel, Shal-related subfamily, member 1; prominent in the repolarization phase of the action potential	Seizures
*GRIPAP1* (300408)	GRIP1 associated protein 1; dendritogenesis, synaptic vesicle release, AMPA receptor exocytosis	Seizures
*PRAF2* (300840)	PRA1 domain family, member 2; protein of synaptic vesicle membranes	Seizures
*PLP2* (300112)	Proteolipid membrane protein, colonic epithelium-enriched differentiation-dependent protein A4	ID
*CCDC22* (300859)	Coiled-coil domain-containing protein 22	ID
*IQSEC2* (300522)	IQ motif- and SEC7 domain-containing protein 2; in neurons: cytoskeletal organization, dendritic spine morphology, excitatory synaptic organization	ID, autistic behavior, psychiatric problems, delayed early speech development
*SMC1A* (300040)	Structural maintenance of chromosomes 1A; Cornelia de Lange syndrome type 2	Seizures

Within the framework of diagnostic array CGH testing of a cohort of 448 patients presenting ID, epilepsy, and minor anomalies, we detected overlapping duplications affecting the Xp11.23p11.22 region in three female patients. In two girls, we identified the already known recurrent duplication, while in the third patient a large, non-recurrent copy number gain was detected.

## Materials and Methods

### Subjects

In the Department of Medical Genetics, we collected blood samples from probands with ID, seizures and/or congenital malformations and dysmorphic features, and from their family members. Written informed consent for genetic testing was obtained in genetic counseling from all individuals examined or their guardian, as well as from their healthy relatives. Genomic DNA was isolated from peripheral blood according to standard procedures. This study was performed in accordance with the Hungarian genetic law (XXI/2008).

### Patient 1

She is the first child of non-consanguineous parents. The mother has epilepsy and mood disorder; as well the father has mood disorder. Three of the first cousins of the mother are treated with hyperactivity; the maternal grandmother had hearing impairment. One of the father’s first cousins was born from a consanguineous marriage, and had a muscular disorder, without a correct diagnosis. Unfortunately, only the mother was available for genetic testing, the distant relatives were not.

She was born following an uneventful pregnancy in the 39th week of gestation, per vias naturals (birth weight 3,370 g, Apgar scores 9/10). Her early psychomotor development was delayed (determined by Bayley Scales of Infant and Toddler Development); she receives neurohabilitation since her 8 month of age. As a result, she sat at 15 month of age and stood up at the age of 18 months. Delayed language development was detected, at 19 month it is limited to one word. Brain magnetic resonance imaging (MRI) at 16 month of age showed supratentorial abnormalities in the white matter, hypoplasia of corpus callosum, and plexus chorioideus cysts. At 2 years of age, compulsive behavior occurred, with bruxism, hand biting and repetitive hand movements. She does not keep eye contact, and unreasonable laughter can be observed, in addition, her speech is inarticulate. There is a stagnation and slight regression in motor development. Her first epileptic seizure developed at the age of 28 months, appropriate seizure control was achieved by valproate monotherapy (Table 2).

**TABLE 2:** Comparison of clinical features in our patients.

		**Patient 1**	**Patient 2**	**Patient 3**
At birth	Weeks of gestation	39th	42nd	33rd
	Birth weight	3,370 g	2,900 g	1,480 g
	Apgar scores	9/10	9/10	N/A
Last examination	Age	1, 5year	13 years	19 years
	Weight	8,620 g (<3 percentile)	75 kg (>97 percentile)	64 kg (50–75 percentile)
	Height	82 cm (50 percentile)	165 cm (75–90 percentile)	150 cm (< 3 percentile)
	Head circumference	43, 5 cm (<3 percentile)	N/A	54, 5 cm (25–50 percentile)
Dysmorphism	Microcephaly	+	−	−
	Biparietal diameter	−	−	decreased
	Flat occiput	+	−	−
	Low frontal hairline	+	−	−
	Low posterior hair line	−	+	−
	Flat face	+	+	−
	Round face	−	+	−
	Thick eyebrows	+	−	+
	Synophrys	+	+	−
	Eyelids	Anti-mongoloid	−	Mongoloid
	Hypertelorism	+	−	−
	Asymmetrical eyes	−	Smaller eye on the left side	−
	Short philtrum	+	+	−
	Wide nose	−	−	+
	Downward corner of the mouth	−	+	−
	Retromicrognathia	+	−	−
	Prominent mandible	−	+	−
	Low set ears	+	−	−
	Short neck	−	+	−
	Hands	Thick fingers, brittle nails	Mild syndactyly on fingers II-III-IV, tapering fingers	Clinodactyly of the 5th fingers on both sides
	Feet	−	−	Lateral deviation of the first toes on both sides
	Other	Joint laxity, hypertrichosis	Hypoplasia of the labia minora	A hemangioma capillare of 5 cm in diameter above the left elbow

She was temporarily cared by an ophthalmologist because of divergent strabismus. She often had upper respiratory infection and inflammation of the eyes. The sequencing of *MECP2*, *FOXG1*, *CDKL5* genes gave normal results.

### Patient 2

She was born from the first, uncomplicated pregnancy of her mother with cesarean section from meconium-stained amniotic fluid. The mother’s sister has short stature, small feet, she is slow moving, with an IQ at the lower limit of normal and similar facial characteristics as our Patient 2 (The sister did not consent to genetic testing).

In the background of feeding difficulties and delayed development of this patient generalized muscle hypotonia was detected at the age of 8 months. As a result of neurohabilitation therapy she walked alone at 28 months of age. Her speech development was severely delayed; she used short sentences from the age of 5–6 years with articulation errors (Budapest-Binet Intelligence Scale). Now she attends a special education school. She has many friends, helps at home and loves to play with a ball. According to the parents, she would be aggressive if not handled well. Brain MRI at age of 1 year showed cerebral atrophy, subdural hygroma, and parietally on the right side a small demyelinated focus was displayed. Three years later the control MRI detected discrete supratentorial, subcortical white matter abnormalities. Weight gain started around the age of 3 years due to compulsive eating, she has regular endocrinological surveillance due to her obesity. Laboratory investigations excluded Prader-Willi syndrome (Table 2).

### Patient 3

She was born at the 33rd week of gestation with a weight of 1,480 g after premature rupture of membranes. She was adopted; no family history can be obtained except that her mother has intellectual disability as well. In the perinatal period, she was treated for hypoglycemia, omphalitis, hyperbilirubinemia, and urinary tract infection. Gastroesophageal reflux was confirmed in the background of apnea. Her early psychomotor development was delayed. She was never toilet trained. Her first epileptic seizure developed at the age of 10 years, the EEG (electroencephalography) examination showed fronto-temporal epileptic discharges on the right side. She has been seizure-free with lamotrigine monotherapy for 2 years. The brain MRI detected no abnormalities. Aggression, tantrums have been observed since childhood, risperidone was applied. She attends a special school because of the moderate intellectual disability (tested by Budapest-Binet Intelligence Scale). Regression has been noticed at some developmental areas. Menarche occurred at 10 years of age, the menses is irregular (Table 2).

### GTG Banding

Karyotyping from cultured peripheral blood lymphocytes was performed by Giemsa–Trypsin (GTG) banding at 550 bands per haploid set using standard procedures (Caspersson et al., 1970).

### Array CGH

Array CGH was performed using Agilent Human Genome Unrestricted G3 ISCA v2 Sureprint 8 × 60K oligo-array (Amadid 021924) (Agilent, Santa Clara, CA) (Kallioniemi et al., 1992). DNA was isolated from peripheral blood leukocytes using the NucleoSpin^®^Dx Blood DNA Purification Kit (Thermo Fisher Scientific, Waltham, MA) as recommended by the manufacturer. For calculation of the concentration and purity of the isolated DNA NanoDrop spectrophotometer was used. Labeling and hybridization of the samples was made according to the Agilent Oligonucleotide Array-Based CGH for Genomic DNA Analysis—Enzymatic Labeling Protocol. Washing was performed following the instructions of Agilent Protocol v7.2. The results were obtained by Agilent dual laser scanner G2565CA and processed with Agilent Feature Extraction software (v10.10.1.1.). Agilent Cytogenomics software (v4.0.1.) was used for evaluation of the CNVs. DNA sequence information refers to the public UCSC database (Assembly: Human GRCh37/hg19). The CNVs detected were compared to known aberrations available in public databases like DECIPHER (Database of Chromosomal Imbalance and Phenotype in Humans using Ensembl Resources), the Database of Genomic Variants, Clingen Dosage Sensitivity Map, Clinvar, and Ensembl (among others).

### X-Inactivation Study

For determination of the X-chromosome inactivation pattern the human androgen receptor gene (*AR*) assay (HUMARA) was performed on peripheral leukocytes (in case of Patient 1 and her mother, Patient 2 and her parents, and Patient 3 without family members). The assay is based on PCR analysis of the polymorphic CAG repeat containing region of the *AR* gene, comparing the pattern of DNA samples digested with *Hpa*II methylation sensitive restriction enzyme to undigested samples (Allen et al., 1992).

## Results

### G-Banding

The karyotype of Patient 1 showed a duplication on the short arm of chromosome X. Chromosome analysis of the mother revealed the presence of the same duplication on one of the X chromosomes. In Patients 2 and 3 the laboratory investigations resulted in a normal female karyotype at 550 bhps resolution.

### Array CGH

The breakpoints and sizes of the duplications of chromosome X are the following (according to McGowan-Jordan et al., 2016): Patient 1: arr[GRCh37] Xp11.4p11.21(39969653_58051765) × 3, which means a 18,082 kb duplication; Patient 2: arr[GRCh37] Xp11.23p11.22(46994270_52693966) × 3, with the size of 5,700 kb; and Patient 3.: arr[GRCh37] Xp11.23p11.22(48584351_51956858) × 3, a copy number gain of 3,373 kb, respectively. In addition, common benign variants were detected in all three of the patients. The base pair positions of the genomic imbalances refer to the February 2009 Assembly (GRCh37/hg19). In summary, duplication is of maternal origin for Patient 1 and *de novo* for Patient 2. In case of Patient 3 none of the parents were available for genetic testing.

### X-Inactivation Study

The human androgen receptor assay detected random X-inactivation pattern in Patient 1 and in her mother as well. The results of the test showed similarly random X-inactivation in Patient 2. In contrast, the assay detected non-random X-inactivation pattern in Patient 3 with the same X-chromosome being preferentially inactivated in each of the cells. Unfortunately, in absence of parental samples the origin of the active X chromosome cannot be determined.

## Discussion

Studying the literature data on Xp11.22p11.23 duplication, several interesting observations emerge. Although a limited number of such cases have been reported so far, it can be seen that the results of each recent study are inconsistent with one of the previous observations. However, in spite of the size and gene content differing among previously reported patients with Xp11.22p11.23 duplications; it is our opinion that the clinical symptoms are similar. Attempts to link certain symptoms to one or a few genes are remarkably ineffective in this patient group.

Based on a comparison of our three patients and the cases published so far, developmental delay, intellectual disability with varying severity, seizures and different behavioral abnormalities are the most common major symptoms (Supplementary Table 1). This is not surprising, as this genomic region contains a number of genes associated with ID, from which *SHROOM4, DGKK, KDM5C, IQSEC2, HSD17B10*, and *HUWE1* are included in most publications (Table 1). Based on these features, Xp11.22p11.23 duplication could be classified as neurodevelopmental disease. These symptoms are all present in the three patients described here, although the extent of their duplication varies significantly.

To our knowledge, regression has been described rarely in similar patients so far. We observed it in Patients 1 and 3, especially in the field of motor skills. In a male patient with recurrent Xp11.22p11.23 duplication, Flynn et al. (2011) described regression in areas of speech, memory and recognition, furthermore, leading to aggressive behavior. Helm et al. (2017) reported regression of development with co-occurrence of the onset of seizures in a 20 year-old male patient. Until now, we do not know the background of this symptom either.

Behavioral abnormalities are also characteristic of our three patients: compulsive behavior with bruxism, hand biting and repetitive hand movements, no eye contact and unreasonable laughter (Patient 1), aggression (Patients 2 and 3) and tantrums (Patient 3). The authors of studies on Xp11.22p11.23 duplication in the context of autistic behavior and attention deficit and hyperactivity raise the role of *KDM5C*, *SHROOM4* and *IQSEC2* genes (Table 1). Our Patients 1 and 2 present such symptoms, in spite of *SHROOM4* being present in both of them (and in Patient 3 without similar symptoms), however, *KDM5C* and *IQSEC2* genes are involved only in the duplication of Patient 1. Within the area of Xp11.22p11.23 duplication, in the cases studied so far, two genes have been associated with behavioral abnormalities, *SYN1* and *ZNF674* (Table 1). Both genes are duplicated in case of Patient 1, only *SYN1* is affected in Patient 2. However, all three of our patients struggle with some kind of behavioral disorder.

Epilepsy is an important symptom of the disease; it has been described in about half of the cases reported so far (Giorda et al., 2009; Holden et al., 2010; Edens et al., 2011; Nizon et al., 2014; Grams et al., 2015). Seizures occurred in two of the three patients studied by us (Patient 1 and 3). Table 1 contains 11 genes that may play a role in epilepsy.

It can be seen from the data of Supplementary Table 1 that the somatic parameters of the patients with Xp11.22p11.23 duplication vary widely. Higher than average values for head circumference (OFC) can be seen as typical. Our most noteworthy observation on Patient 1 refers to her OFC (below the 3rd percentile). As far as we know, only two girls has been reported to date with OFC < 3rd percentile (Patient 2 at Edens et al., 2011; Arican et al., 2018). In addition, the mother of Giorda’s Patient 1 should be mentioned with the same OFC value. Examining the role of the underlying genes, we can see that *PQBP1* has been associated with microcephaly. One might also speculate that for our Patient 1, it may be explained by the large duplication. However, Patient 1 by Edens et al. (2011) and that of Holden et al. (2010) have large, overlapping duplications also, nevertheless, OFC is at 75th and 90–97th percentile, respectively. The examples listed above demonstrate that studying the size and gene content of Xp11.22p11.23 duplications alone does not provide an explanation for these differences.

Brain MRI showed aspecific abnormalities in a number of reported cases including our cases: Patient 1 and 2 share the supratentorial white matter abnormalities, in addition we observed even corpus callosum hypoplasia and plexus chorioideus cysts in Patient 1., and subdural hygroma in Patient 2 with a small demyelinated focus on the right side.

Duplication of Xp11.22p11.23 is accompanied by dysmorphism in most cases known to date, but the features observed in individual patients are not specific (Table 2). However, it is worth mentioning synophrys and bushy eyebrows, which are present in about half of the patients, including the three girls reported here (Supplementary Table 1 and on the photos by Nizon et al., 2014).

In Patient 1 and 2, eye abnormalities as divergent strabismus (Patient 1), and asymmetrical eyes with smaller eye on the left side (Patient 2) have been also observed. Small eyes are characteristic also for Patient 9 at Grams (2015). In the same study myopia were described at Patient 3 (and in her mother), cataracts, in Patent 5 pseudostrabismus, hyperopia and astigmatism. Early onset myopia and bilateral hypopigmentation of the midperiphery were reported by Zou and Milunsky (2009). Hypermetropic astigmatism occurred in Patient 1 and 2, and recurrent uveitis in Patient 3 described by Giorda et al. (2009). These examples demonstrate that eye disorders are not uncommon in patients with Xp11.22p11.23 duplication, however, only four genes as *BCOR*, *NDP*, *NYX*, and *RP2* are known associated with abnormalities of the eyes and these are involved in the duplication of our Patient 1 and the case reported by Zou and Milunsky (2009). The further above mentioned patient’s eye abnormalities cannot be explained by the extra copy of these four genes.

A similar conclusion can be obtained when examining the symptom of early puberty described in about 50% of the patients. As far as we know, *BMP15* is the only gene associated with early puberty. *BMP15* is affected in all three of our patients, but this symptom is present in two of them only (for similar cases see Supplementary Table 1). Only two patients are listed in Supplementary Table 1 where the duplication does not affect this gene: the 3rd patient reported by Grams et al. (2015) and the case described by Honda et al. (2010). While in the latter case the author did not report data on early puberty, this symptom was described in the patient of Grams et al. (2015). Based on our three patients, age could be an explanation, as Patient 1 not affected by early puberty, is very young. This theory could be applied to 4, 5, and 8th patients of Nizon et al. (2014) (4, 5, and 6 years old girls, respectively) as well. However, Supplementary Table 1 also contains some older patients with *BMP15* duplication without early puberty (e.g., 4th patient of Giorda and 9th patient of Grams, both of them with 14 years). Therefore, an extra copy of the *BMP15* gene alone may not be a sufficient explanation for the development of this symptom, although reduced penetrance should also be considered.

To examine the causal relationship between Xp11.22p11.23 duplication and phenotype, several factors need to be considered. Which genes and regions may play a role in the development of the symptoms, and which factors may affect the expression of these genes (regulatory elements—primarily *cis*-acting elements with respect to duplication, the copy number of affected genes, and closely related to this the active functioning copy number influenced by X inactivation).

Among the cases published so far, duplications of various size occur. Therefore, it is difficult to identify a critical region. The most experimental evidence support the role of Region 1 and 2 reported by Grams et al. (2015) (see above). The fact that the known recurrent and non-recurrent Xp11.23 duplications involve these regions is definitely an argument for it (Figure 1). It is an interesting observation that the patient reported by Honda et al. (2010) is the only one shown in Figure 1 whose duplication does not affect these regions. She shows relatively fewer symptoms, mostly intellectual disability that is associated with more genes (see Table 1) being involved in her duplication. Particular attention should be paid to cases where duplication does not overlap with the critical region, but the phenotype does not differ from that of patients with critical region involvement, for example the girl reported by Zou and Milunsky (2009) (see Figure 1). This observation raises the possible role of other factors influencing gene expression, like cis-acting regulatory elements. We still have little information about these for this region now. In any case, studying the ENCODE project data for the genomic region shown in Figure 1, it is striking that H3K27Ac marks are located near the breakpoints of the recurrent duplication which play a role in transcription activation. Based on all this, the genomic region responsible for the phenotype features is most likely to fall into the area of recurrent duplication defined by Giorda et al. (2009).

The occurrence of the Xp11.22p11.23 duplication is well known in both genders, thus, many affected females are described in the literature (e.g., Monnot et al., 2008; Zou and Milunsky, 2009; Holden et al., 2010; Chung et al., 2011; Edens et al., 2011; Evers et al., 2015). According to the authors of Xp11.22p11.23 duplication articles, the cause of the abnormal phenotype is the functional disomy of the genes affected by duplication. This seems to be clear in the case of affected males; however, determination of actually functioning copy number in the female patients is more difficult due to X-inactivation. Not all of the published reports provide data for X-inactivation but in about half of the cases studied, a skewed X-inactivation with preferential inactivation of the normal X chromosome in the majority of the cells was found. In these females, functional disomy is an acceptable causal factor as well. Along this argument, one would expect that females with random X-inactivation show milder clinical features but this is not always the case (Evers et al., 2015; for examples of random X-inactivation see Supplementary Table 1). When interpreting X-inactivation data, it should be taken into account that peripheral blood cells are always examined, and the pattern of X-inactivation may vary from tissue to tissue. An additional challenge for genotype-phenotype analysis is that duplication of Xp11.22p11.23 does not occur exclusively in females with abnormal phenotype. Asymptomatic carrier mothers are reported in some familiar cases, they may have affected offspring of both genders (Giorda et al., 2009; Honda et al., 2010; Grams et al., 2015). In these cases, the normal phenotype does not always involve preferable inactivation of the duplicated X chromosome, i.e., skewed X inactivation does not correct for the effect of duplication. This phenomenon suggests the role of additional regulatory factors also. Among our cases, Patient 1 and her mother who has the same Xp11.22p11.23 duplication have random X-inactivation pattern. Given that the phenotype of Patient 1 is much more severe compared to her mother who has only mild intellectual disability, and the random X inactivation suggests similar active functioning copy number, we must also assume the role of further factors besides the copy number of the duplicated genes. However, the most severe phenotype can be seen in Patient 3 with non-random X-inactivation which reinforces the pathogenic role of elevated copy numbers of the genes involved later on.

## Conclusion

The duplication of the p11.22p11.23 region of the short arm of X chromosome, as well as its effect on the phenotype is known from the description of only a limited number of cases to date. The detailed description of the three patients we studied contributes with new observations to the clinical data that have become known so far related to this rare disease. The recognized cases have mostly been examined in diagnostic centers, where the examination possibilities are limited, which in addition to rare occurrence of this abnormality, may also be the reason why very little is currently known about the relationship between genotype and the resulting phenotype. As the comparison of our patients with others reported to date clearly demonstrate, that in addition to the breakpoints of the duplication and the role of the genes involved, a number of other factors influencing gene expression may affect the symptoms that appear.

Deciphering of the secret can be hoped for from systematic analysis of the genomic data, the gene expression, X-inactivation in multiple tissues, as well as other factors involved in the regulation of gene expression. This rare disease with its peculiarities offers an opportunity to gain insight into the functioning of this section of the X chromosome, primarily into the role of regulatory factors and mechanisms.

## Data Availability Statement

The raw data supporting the conclusions of this article will be made available by the authors, without undue reservation.

## Ethics Statement

Ethical review and approval was not required for the study on human participants in accordance with the local legislation and institutional requirements. Written informed consent to participate in this study was provided by the participants’ legal guardian/next of kin.

## Author Contributions

JZ and ÁT were responsible for the patient’s clinical genetic examination. AZ contributed to the clinical description. AM, MC, and AS were responsible for methodology and software analysis. KH and ÁT made the conceptualization of the study. ÁT and MC prepared the writing—original draft. KH and BM reviewed and edited the manuscript. All authors contributed to the article and approved the submitted version.

## Conflict of Interest

The authors declare that the research was conducted in the absence of any commercial or financial relationships that could be construed as a potential conflict of interest. The handling editor KK declared a past collaboration with several of the authors MC, T, AS, AM, BM, and KH.
